# Sound waves of progress: advancing endoscopic ultrasound in pediatric gastroenterology

**DOI:** 10.3389/fped.2025.1634232

**Published:** 2025-08-18

**Authors:** Sumith Roy, Rachel Gitter, Jennifer Nguyen, Andrew Huang, Clifton Huang

**Affiliations:** ^1^ Department of Pediatric Gastroenterology, Cook Children’s Health Care System, Fort Worth, TX, United States; ^2^ Department of Pediatrics (Pediatric Research Program), University of North Texas Health Science Center, Fort Worth, TX, United States; ^3^ Department of Pediatric Gastroenterology, University Hospitals Rainbow Babies & Children’s, Cleveland, OH, United States; ^4^ Department of Pediatric Gastroenterology, University of Nebraska Medical Center and Children’s Hospital and Medical Center Omaha, Omaha, NE, United States; ^5^ Department of Pediatrics, Burnett School of Medicine at TCU, Fort Worth, TX, United States

**Keywords:** EUS, endoscopic ultrasound (EUS), radial EUS, linear EUS, choledocholithiasis, pediatric gastroenterologist

## Abstract

**Introduction:**

A substantial body of research validates the application of endoscopic ultrasound in adults in the management of biliary and pancreatic conditions. There is limited data regarding its impact on children, although its use has steadily increased over the years. This study aims to assess long-term data on indications, efficacy and safety of endoscopic ultrasound in pediatric patients.

**Methods:**

This ambispective registry data reflects a single-center experience of endoscopic ultrasound either as a stand-alone procedure, or in conjunction with guided interventions such as lumen-apposing metal stents or as a pre-assessment tool prior to other interventions such as endoscopic retrograde cholangiopancreatography, both of which were independently performed by a pediatric gastroenterologist.

**Results:**

A total of 294 EUS were performed in 216 patients (55.1% female, median age (IQR) 15 [11.9–16.9] years). Technical success was 100% and no complications were associated with EUS.

**Discussion:**

This robust dataset adds to the emerging evidence of EUS performed with a high technical success rate coupled with low risk for adverse events.

## 1 Introduction

Endoscopic ultrasound (EUS) is an advanced endoscopic tool that has an incorporated ultrasound probe allowing for the visualization of individual layers of the gastrointestinal (GI) tract as well as adjacent organs such as liver, biliary tract and pancreas. A robust body of research supports the use of EUS in adults for various diagnostic and therapeutic indications (1). The utilization of EUS in pediatric GI conditions has expanded over the years; however, nuances remain in its use in children in the absence of established diagnostic criteria. Additionally, performing these advanced procedures in children can be challenging due to their small anatomy. Currently, standard radial and linear echoendoscopes are used in children, limiting their use in patients less than 13 kg. In Barakat et al. assessed the pediatric endoscopy practice patterns in North America, Canada and Mexico. The authors reported that, out of the sixty-three pediatric program respondents registered with the North American Society for Pediatric Gastroenterology, Hepatology and Nutrition, 46.5% of the institutions performed EUS. Among those institutions, adult gastroenterologists performed approximately 75.8% of the procedures. Additionally, 5.0% of the respondents reported greater than ten EUS procedures performed annually (2). More recently, pediatric endosonographers Piester and Liu described the technical success and outcomes of EUS in ninety-eight pediatric patients (3). In the present study, we investigate the safety and efficacy and the clinical outcomes of pediatric patients who underwent EUS as part of their disease management.

## 2 Methods

This ambispective study utilized data collected as part of the EUS Research Registry, including 216 patients undergoing EUS at Cook Children's Healthcare System from March 1, 2023 to March 31, 2025. Inclusion criteria were all male and female patients from birth to 21 years of age (inclusive) in whom EUS was performed, as deemed necessary by the treating physician. Information regarding demographics, pre and post procedure evaluations (including, clinical, laboratory, imaging), EUS procedure details were recorded. EUS-specific American Society of Gastrointestinal Endoscopy guidelines were used to document occurrence of adverse events (4). Follow up data was collected, for (1) all patients to assess if there were any intra procedural or immediate post-op adverse events (2) for patients requiring a repeat EUS. Repeat EUS was done in patients with hereditary pancreatitis who presented with chronic pancreatitis, stone retrieval, stricture management, need for stent revision/replacement requiring subsequent interventions such as endoscopic retrograde cholangiopancreatography (ERCP). EUS was performed using Olympus radial echoendoscope (GF-UE 160), Olympus curvilinear echoendoscope (GF-UCT 180) and the Olympus Ultrasonic miniprobe system (UM-3R) [Olympus America, Inc. Center Valley, PA, USA]. Data was managed using Research Electronic Data Capture (5).This study received Institutional Review Board approval. A pediatric gastroenterologist trained in advanced endoscopy independently performed all procedures. Technical success of the procedure was defined as the ability to complete the intended procedure with no adverse events and complications following the procedure.

### 2.1 Statistical analysis

Descriptive statistics were calculated to summarize patient characteristics and were reported based on normality. All statistical tests were two-sided and a *p*-value less than 0.05 was considered as statistically significant. Data was analyzed in SAS Enterprise (version 8.3; SAS Institute Inc., Cary, NC, USA).

## 3 Results

A total of 294 EUS procedures were performed at this single center on 216 pediatric patients from March 1, 2023 to March 31, 2025. EUS was performed with a 100% success rate. Table 1 outlines the demographic characteristics. All procedures were performed under general anesthesia. Out of the 216 patients undergoing EUS, eight patients weighed less than 15 kg, in whom EUS miniprobe were used through a 2.8 mm working channel of the endoscope. The youngest child to receive an EUS miniprobe was an 8-week-old infant weighing 4.5 kg presenting with signs and symptoms suggestive of esophageal stricture/fibrosis. The youngest patient to receive radial EUS was 22 months of age and weighed 11.7 kg to assess esophageal wall thickness for suspected eosinophilic esophagitis.

**Table 1:** Demographic characteristics of patients in whom EUS was performed.

Patient characteristics (*N* = 216)	*N* (%)
Age [median, years (IQR)]	15 [11.9–16.9]
Sex	
Female	119 (55.1)
Male	97 (44.9)
Race	
White	172 (79.6)
Black or African American	29 (13.4)
Asian	11 (5.1)
Other	4 (1.9)
Ethnicity*	
Non-Hispanic	126 (58.6)
Hispanic	89 (41.4)
Weight (median, kg [IQR])	63.2 [39.3–81.5]
<15 kg	8 (3.7)

* *n* = 215 (missing data on 1 patient).

The indications of EUS (Table 2) are described below categorized based on diagnostic and therapeutic applications:

**Table 2:** EUS indications by organ system.

Indications	Procedures *N* (%)
Biliary/Hepatic	148 (50.3)
Pancreatic	64 (21.9)
Esophageal	44 (15.0)
Rectal	16 (5.4)
Subepithelial/submucosal lesions	6 (2.0)
Other	16 (5.4)
**Total**	**294**

### 3.1 Diagnostic indications

#### 3.1.1 Biliary/hepatic

**Figure 1:**
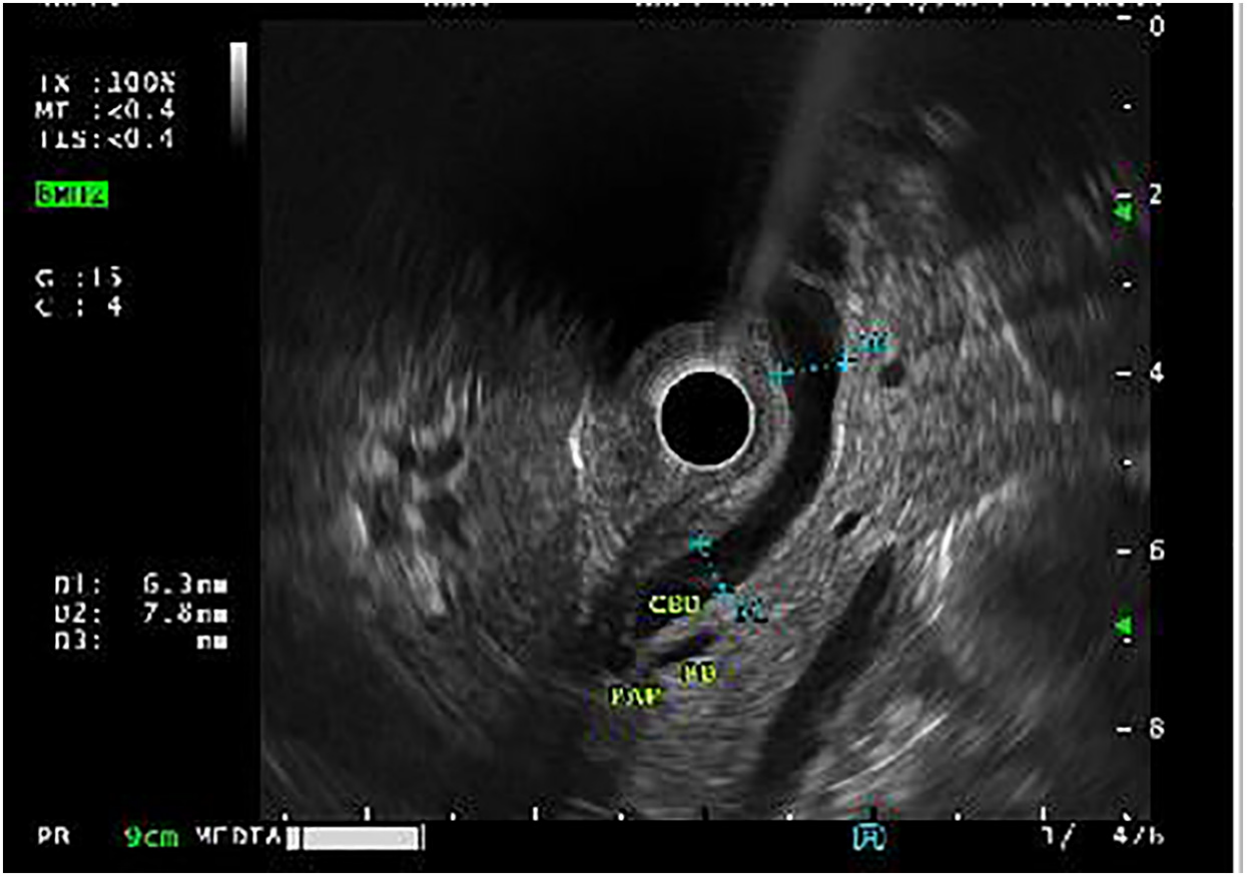
Endoscopic ultrasound (EUS) demonstrating absence of choledocholithiasis, averting endoscopic retrograde cholangiopancreatography (ERCP).

A total of 148 (50.3%) EUS procedures were performed for biliary/hepatic indications. Among them, 100 (67.6%) were performed for suspected choledocholithiasis in whom ultrasound sonography (USG) or Magnetic resonance cholangiopancreatography (MRCP) were inconclusive. Performing an initial EUS, averted a total of 10 (10%) ERCPs (Figure 1).

Of the 93 patients with choledocholithiasis, 42 met criteria for obesity, defined as body mass index greater than the 95th percentile according to sex-specific CDC growth charts for age (6). Ten patients had hemolytic disorders associated with an increased risk of choledocholithiasis. A total of 15 patients experienced symptoms along with elevated lipase levels (greater than 4 times the upper limit of normal) suggestive of gallstone pancreatitis. The median common bile duct diameter was 6 mm (IQR: 5–8.1). Typically, patients undergo EUS and/or ERCP prior to cholecystectomy. Five patients presented with persistent post-cholecystectomy abdominal pain (without prior ERCP). This warranted further exploration with EUS and ERCP for evaluation of stone in the common bile duct. Firth's logistic regression was used to assess significant predictors of need for ERCP, comparing the ERCP averted group and the group who underwent ERCP following EUS. However, there were no statistically significant differences observed between the two groups.

#### 3.1.2 Pancreatic

Pancreatic disorders accounted for the second highest indication (21.9%) for EUS. A total of 31 EUS was performed in 11 patients for chronic and/or recurrent pancreatitis. Of the 11, nine had hereditary pancreatitis. Using the Rosemont criteria, patients were assessed for chronic pancreatitis (CP) by EUS (5). Three met criteria for definitive CP and were managed with chronic pain medications, four had findings suggestive of suspected CP, one met criterion for possible CP, and three were classified as normal. Elastography done in four of the eleven patients demonstrated decreased tissue compliance in two patients.

Ten EUS were performed for confirmation and management of pancreatic divisum prior to ERCP, including two in whom MRCP results were discordant (compared to EUS) due to limited visualization, in the setting of pancreatitis.

Trauma-related biliary/pancreatic injury: Fifteen EUS were done for initial evaluation of traumatic biliary (*n* = 8) and pancreatic (*n* = 7) injuries involving motor vehicle collisions, handlebar injuries, and hit-and-run. EUS facilitated precise identification of pancreatic and bile duct diameter thereby providing guidance on stent size placement.

#### 3.1.3 Esophageal

**Figure 2:**
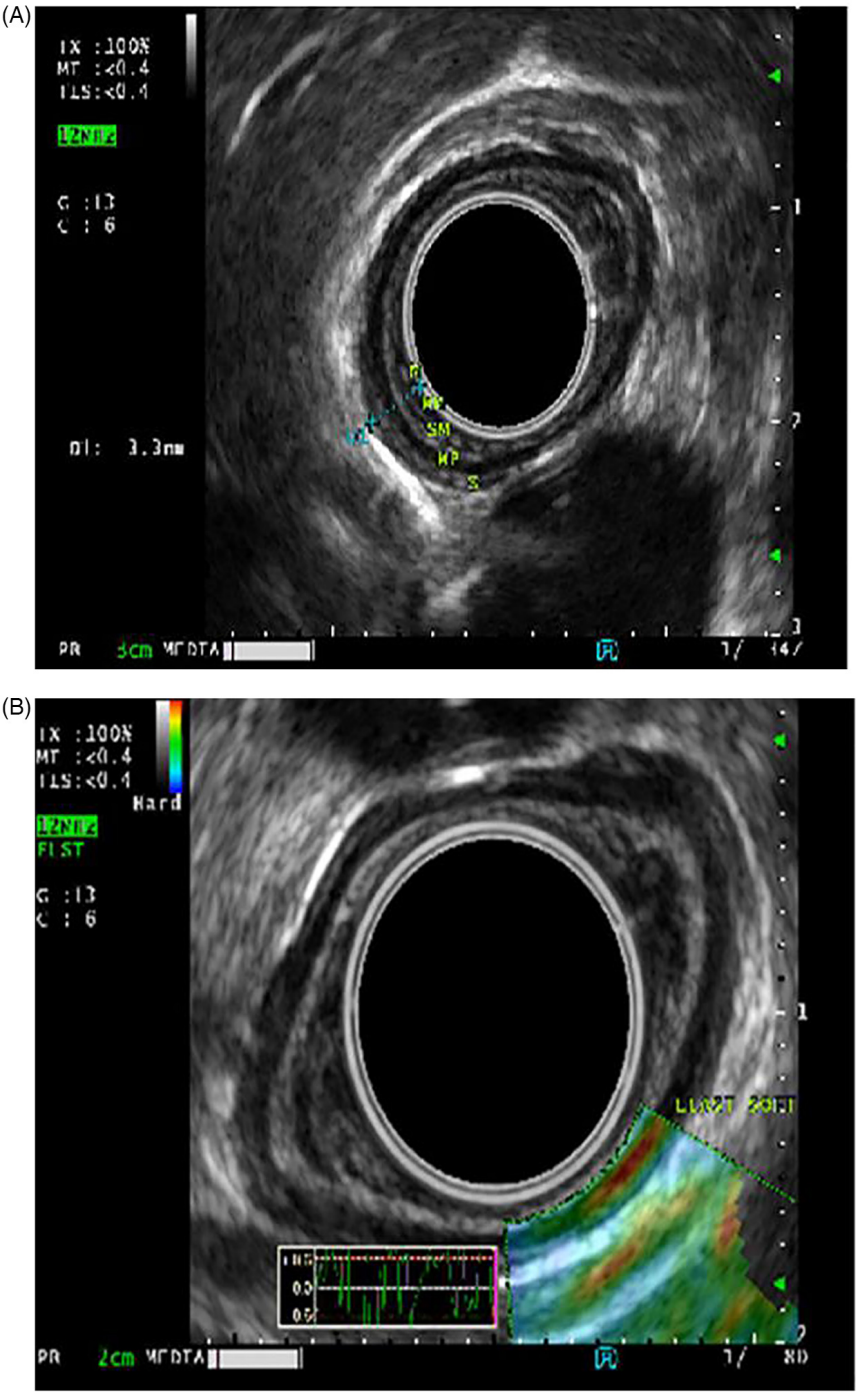
**(A)** Esophageal wall thickness prior to IL-4Rα antibody treatment. **(B)** Elastography demonstrating concerns for esophageal wall stiffness.

EUS was used to evaluate the esophageal wall for long-term complications associated with eosinophilic esophagitis (15 EUS), esophageal strictures (19 EUS) and subepithelial lesions (6 EUS). Esophageal wall compliance was assessed with tissue elastography in eosinophilic esophagitis with suspected fibrosis. Stiffness (by elastography) and fibrosis (histologically) (Figures 2A,B) reverted after 6-month treatment of monoclonal antibody therapy, antibody against IL-4 receptor *α* chain (IL4R*α*) in three patients who had EUS pre and post treatment (Table 3).

**Table 3:** Change in esophageal wall thickness following monoclonal antibody therapy (6–8 weeks post-treatment) .

Patient	Esophageal wall thickness at baseline (mm)	Esophageal wall thickness at follow-up (mm)	Comparative histology at baseline vs. follow-up (eosinophil count/high power field [hpf]) and/or evidence of fibrosis
1	3	1.9	Decrease in eosinophil count from 42/hpf to 3/hpf with resolution of lamina propria fibrosis
2	5.3	2.5	Decrease in eosinophil count from 34/hpf to 9/hpf with resolution of lamina propria fibrosis.
3	2.7	1.8	Decrease in eosinophil count from 45/hpf to normal.

#### 3.1.4 Rectal EUS

**Figure 3:**
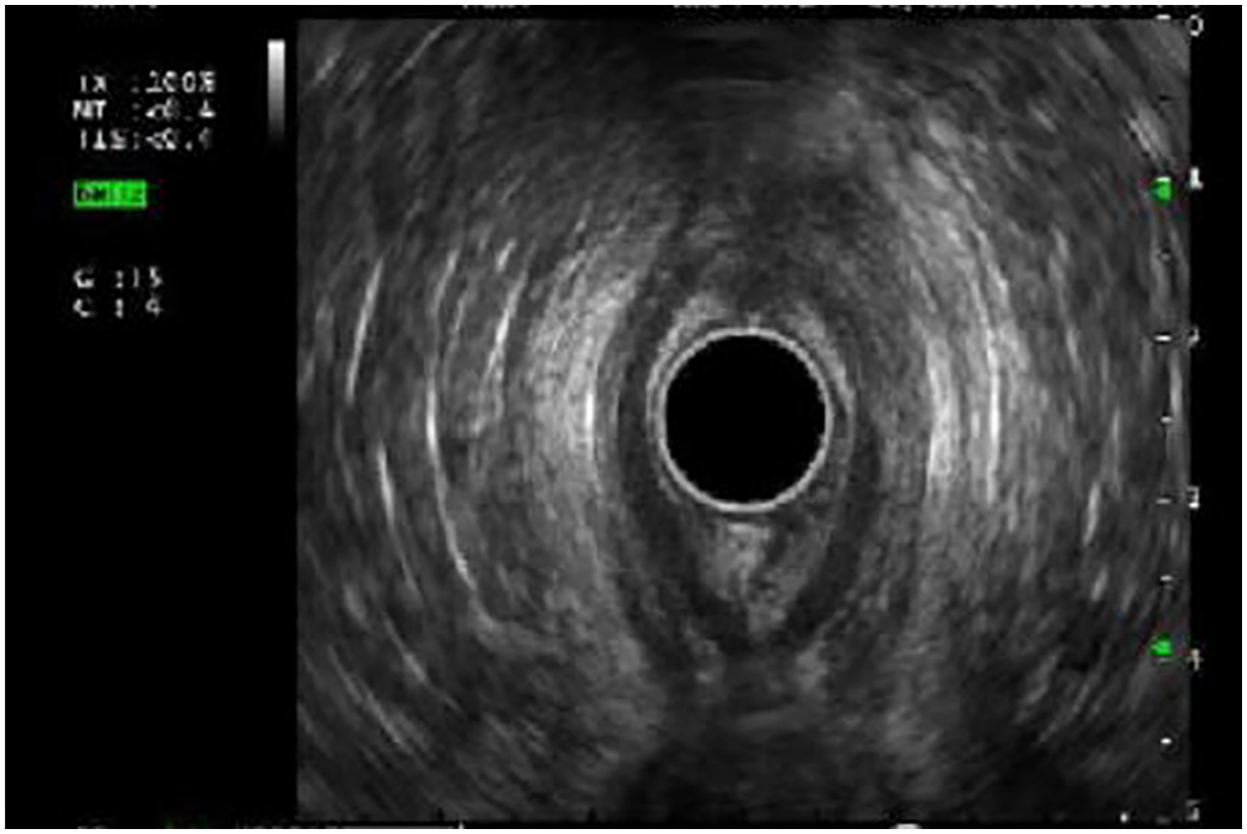
Rectal EUS showing an abscess and fistula at the 12'o clock position.

A total of 16 rectal EUS were performed of which, twelve helped to delineate the extent of perianal involvement (Figure 3) in Crohn's disease. These evaluations are instrumental in determining the need for surgical intervention and optimizing disease management. Among four rectal EUS conducted for suspected malignancy, one involved a fine needle biopsy.

#### 3.1.5 Subepithelial/submucosal lesions

A total of six EUS for subepithelial lesions enabled high-resolution visualization of wall layers, allowing precise determination of the origin of the lesion. Additionally, it helps to determine the need for performing EUS-guided fine needle aspiration/biopsy, thereby enhancing the diagnostic accuracy, risk stratification and management.

#### 3.1.6 Other

A total of 15 EUS were performed on patients who presented with symptoms of chronic abdominal pain, in whom EUS served as an effective diagnostic modality for excluding biliary or pancreatic involvement. Additionally, If ERCP is warranted, it can be performed under the same anesthetic event.

### 3.2 Therapeutic indications

#### 3.2.1 Endoscopic cystogastrostomy for pancreatic pseudocysts

**Figure 4:**
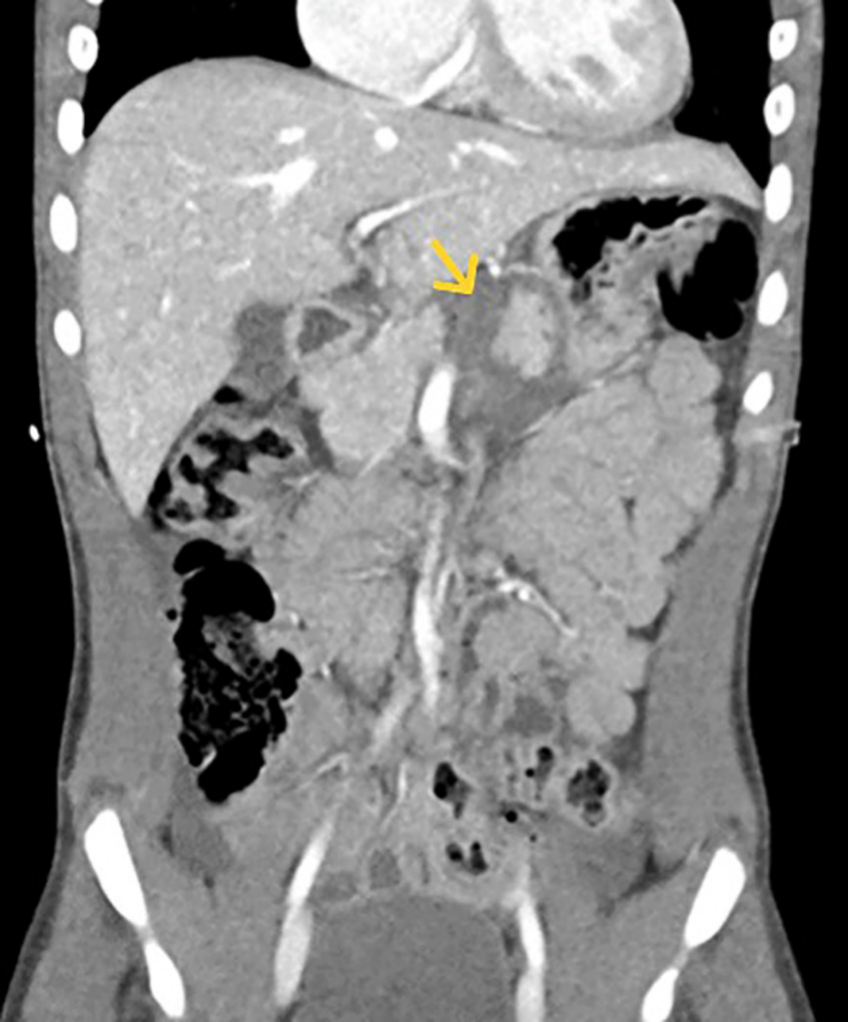
Computed tomography (CT) abdomen demonstrating a pancreatic pseudocyst (indicated by the yellow arrow).

**Figure 5:**
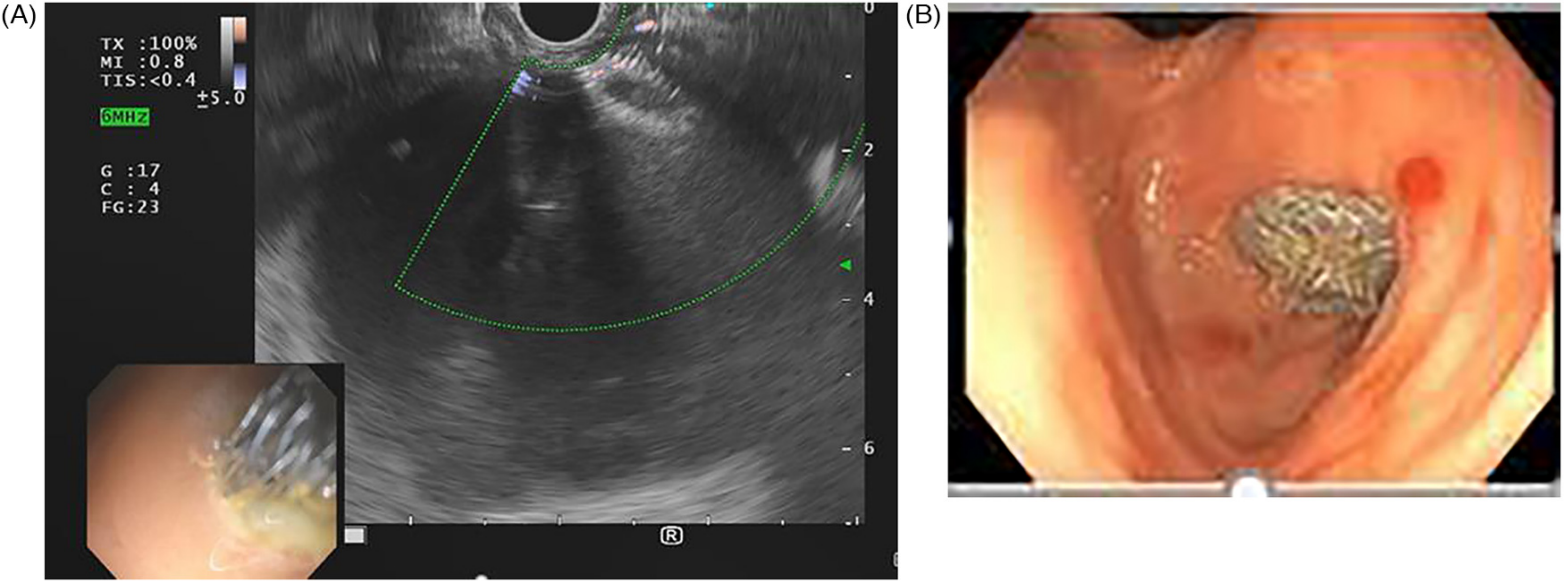
**(A)** Linear EUS during placement of lumen-apposing metal stent (LAMS). **(B)** Endosopic view post-LAMS placement.

Three EUS procedures with lumen-apposing metal stents (LAMS) were performed to manage acute necrotizing pancreatitis with pseudocyst formation (Figures 4, 5A,B) following EUS-guided drainage of pseudocysts, avoiding the need for surgical interventions. Pancreatic necrosectomy was performed using a standard esophagogastroduodenoscopy (EGD), navigating through the AXIOS™ stent (Boston Scientific, Marlborough, Mass, USA) and using biopsy/rat-tooth forceps for the removal of any necrotic pancreatic tissue. Technical success was achieved in 100% procedure.

#### 3.2.2 Refractory esophageal anastomotic stricture

The standard treatment of refractory esophageal anastomotic strictures (EAS) is endoscopic balloon dilation, which temporarily relieves the stricture but can disrupt adjacent normal tissue leading to high incidence of recurrence.

**Figure 6:**
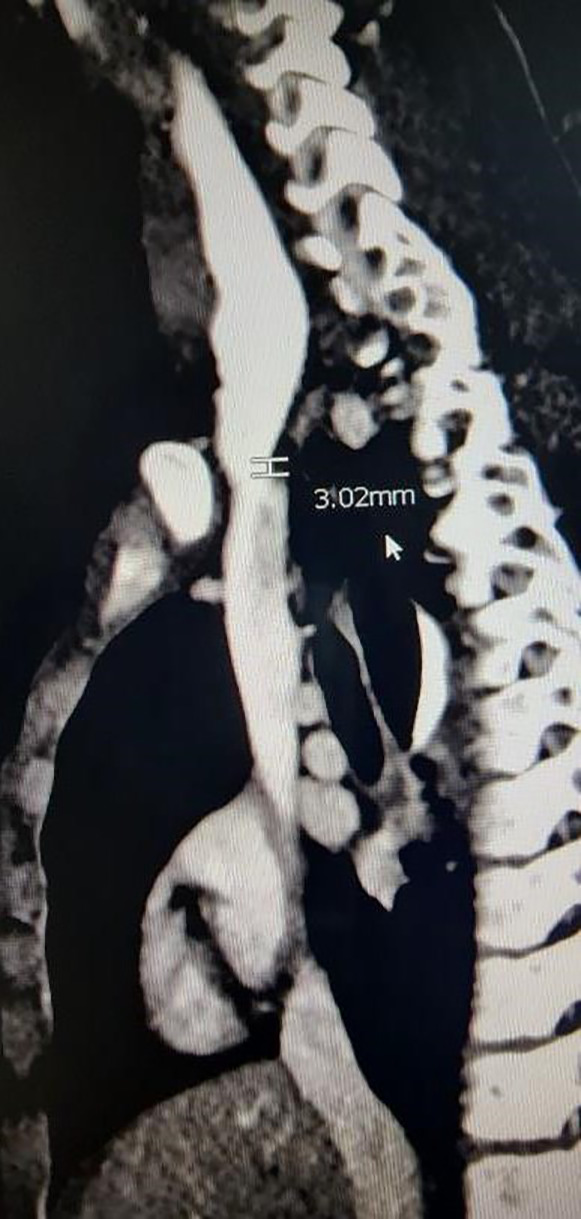
CT chest confirming a short-segment esophageal stricture.

**Figure 7:**
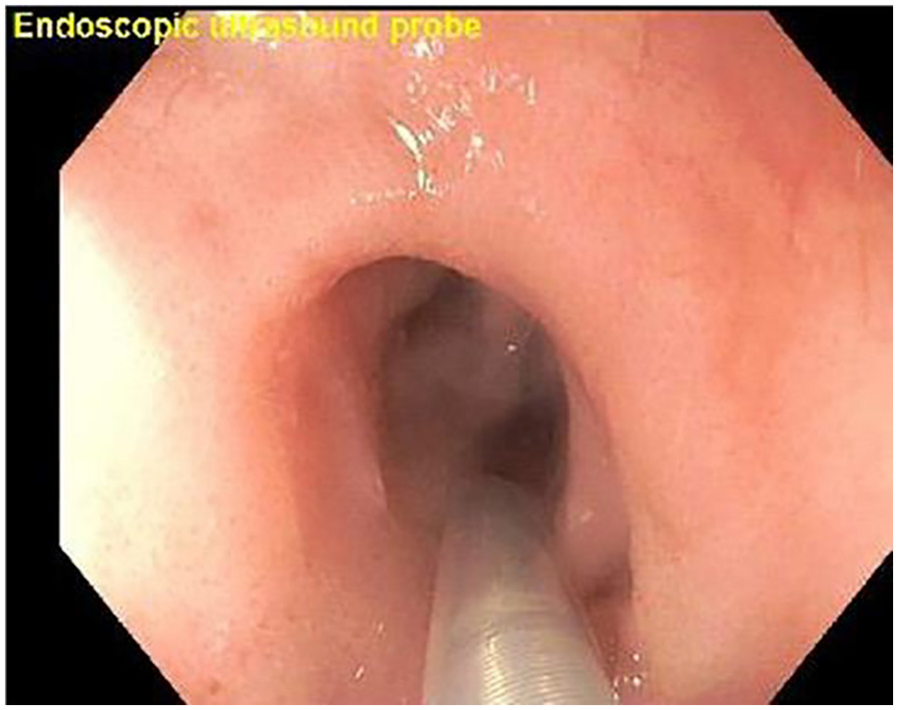
Esophagogastroduodenoscopy (EGD) with EUS miniprobe positioned for high-resolution imaging of the esophageal layers.

**Figure 8:**
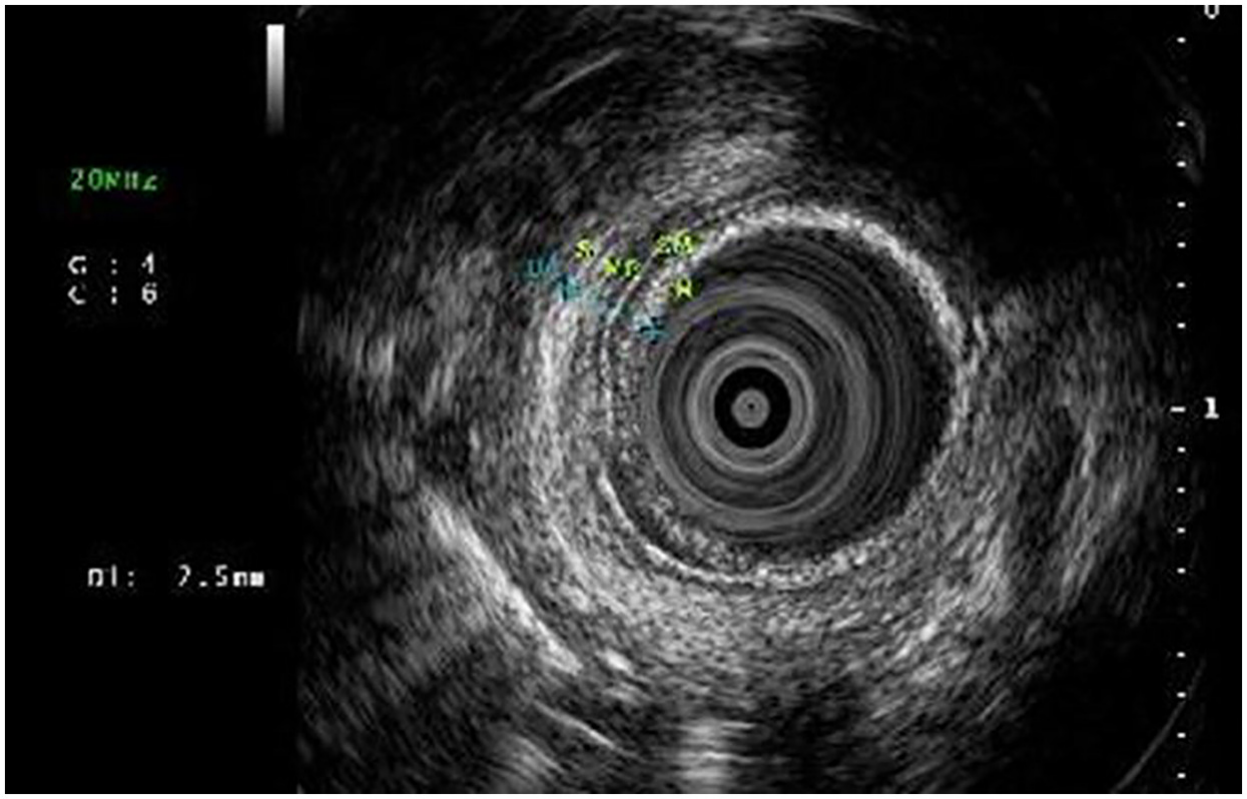
EUS showing axial sections of esophageal layer thickness and potential area of fibrosis.

A refractory esophageal stricture is defined as the inability to maintain a satisfactory luminal diameter (age-appropriate feeding diameter) after a maximum of five dilation sessions with maximal four-week intervals (7). A total of 19 EUS were performed for the management of refractory EAS. Patients underwent EGD, revealing the short-segment anastomotic stricture. Using an EUS miniprobe, wall thickness and length of esophageal anastomotic stricture layers were evaluated. Areas of possible fibrosis were identified by hyperechogenicity and increased wall thickness (Figures 6–8). An esophageal balloon dilator serially dilated the stricture, incrementally (8–18 mm, for 30–60 s per dilation, targeting age-appropriate esophageal diameter under fluoroscopic guidance. Intralesional steroid was administered at the dilated stricture site. There was no evidence of esophageal perforation. A significant decrease in the need for repeat EGD with balloon dilation was observed, with resolution of symptoms at the three -and six-month follow-up.

#### 3.2.3 EUS-guided liver biopsy (EUS-LB)

A total of 28 liver biopsies were performed and the most common indication was abnormal liver function tests (Table 4). The initial 10 patients who underwent EUS-LB were admitted for a twenty-three-hour observation period to monitor for complications, and none were observed. The subsequent 18 patients were discharged two hours after the procedure, once repeat hemoglobin levels were confirmed to be within normal physiological ranges, both of which remain the standard post-operative care at the institution. The mean age was 13.7 ± 3.7 years (50% male). The mean weight was 71.2 ± 22.6 kg. The youngest patient was 4 years old, weighing 15.6 kg.

**Table 4:** Indications for liver biopsy.

Indication	No.
Abnormal liver function test	17
Autoimmune hepatitis	4
Congenital hepatic fibrosis	1
Incidental liver lesion	1
Primary sclerosing cholangitis	3
Wilson's disease	2
Total	28

**Figure 9:**
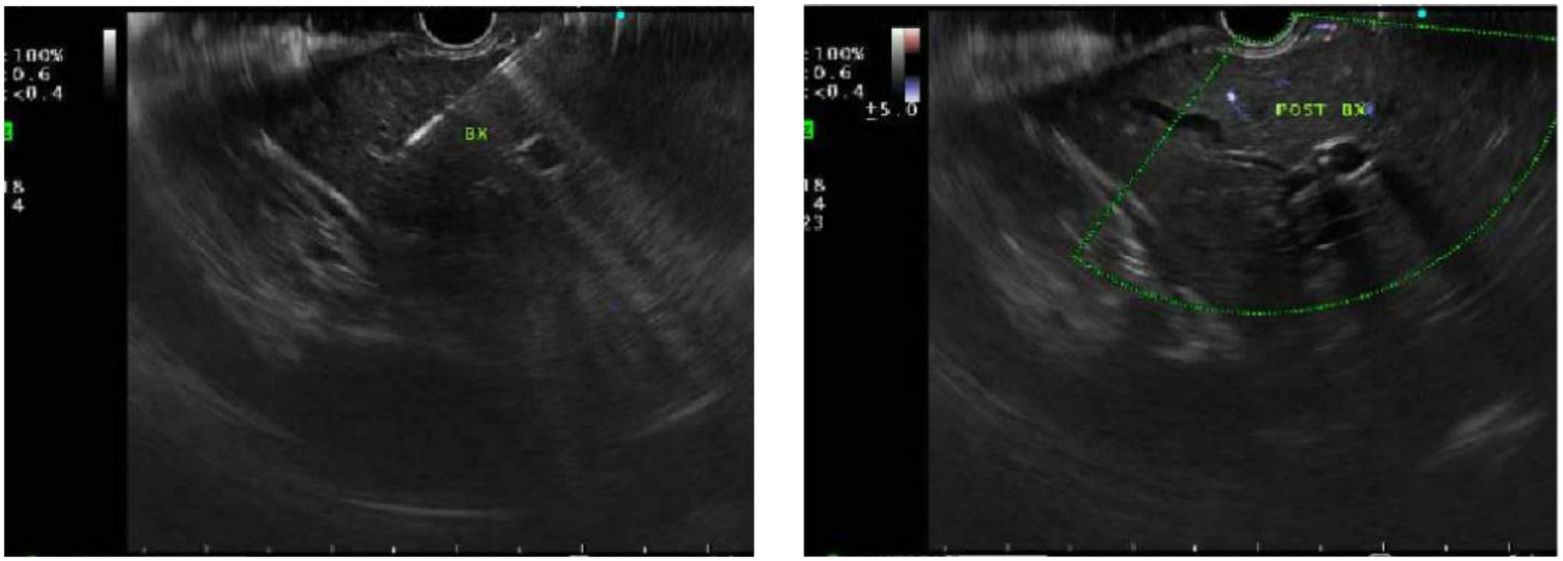
EUS-guided liver biopsy (left) and post-biopsy under Doppler imaging (right).

Biopsies were obtained using a 19G fine needle biopsy without the stylet primed with 10% heparin (wet suction). With the stopcock turned off, the suction syringe was set to −10 ml. A 1–2 actuations/fanning technique was employed (Figure 9). Doppler imaging was first employed to confirm no vascular structures were within the needle path as well as post liver biopsy, to observe for complications such as bleeding. EUS-LB facilitates sampling from both hepatic lobes, thereby enhancing diagnostic yield, and minimizing the need for multiple needle insertions and associated complications. The benefits of performing EUS-LB include same-day discharge and no reported post-procedure pain, thereby enhancing overall patient experience.

## 4 Discussion

Our study represents the highest volume of 294 EUS performed over a twenty-five-month period, which reflects the increased referrals and procedural demand. The predominant indications for EUS in our study involved hepatobiliary and pancreatic assessments, which is comparable to that of existing pediatric and adult literature. The study findings contribute to the growing body of evidence of the utility of EUS in pediatric patients, supporting its safety and efficacy, and highlights absence of complications within our cohort. This is paramount as EUS-guided interventions may reduce the need for more invasive surgical procedures. Moreover, prompt and accurate diagnosis, guided by EUS, potentially improves patient outcomes and long-term prognosis. For instance, EUS proved to be 100% sensitive in our cohort for identifying common bile duct stones. Although the Rosemont classification by EUS is well studied in adults, a similar criterion does not exist in pediatrics. Emerging literature has shown that patients with definitive or suspected CP by Rosemont criteria, were more likely to respond to pancreatic enzyme supplementation (8). Future studies are needed to establish EUS criteria to differentiate pain attributed to chronic pancreatitis from functional abdominal pain.

Some important limitations should be noted in the current study. The data is from a single center and findings might not be generalizable outside of a high-volume tertiary center. Although limitations of the retrospective aspect of the study exists, as in selection bias and ensuring data consistency from historical medical records, we were able to obtain follow-up data and assess clinical outcomes over time.

Emerging technologies include EUS-guided needle-based confocal laser endomicroscopy (9) via transgastric pancreatic puncture, thereby enhancing the diagnostic potential of the CholangioFlex^TM^ (Mauna Kea Technologies, Paris, France) probe in evaluating pediatric pancreatic parenchyma and ductal pathology. Similarly, a transgastric hepatic approach in the management of hepatic parenchyma such as fatty liver disease and autoimmune disease is another promising approach that is yet to be explored. These innovative techniques enable clinicians to tap into molecular imaging strategies to make a timely and accurate diagnosis.

## Data Availability

The raw data supporting the conclusions of this article will be made available by the authors, without undue reservation.
